# Abdominal Aortic Aneurysm and PET/CT: From Molecular Mechanisms to Potential Molecular Imaging Targets

**DOI:** 10.31083/j.rcm2405132

**Published:** 2023-04-27

**Authors:** Chenhao Li, Zhiyin Liu, Gang Yuan, Yong Liu, Weiming Wang

**Affiliations:** ^1^Department of General Surgery (Vascular Surgery), The Affiliated Hospital of Southwest Medical University, 646000 Luzhou, Sichuan, China; ^2^Department of Neurology, The Affiliated Hospital of Southwest Medical University, 646000 Luzhou, Sichuan, China; ^3^The State Key Laboratory of Quality Research in Chinese Medicine of Macau University of Science and Technology, Avenida Wai Long, 999078 Taipa, Macau; ^4^Key Laboratory of Medical Electrophysiology, Ministry of Education & Medical Electrophysiological Key Laboratory of Sichuan Province, (Collaborative Innovation Center for Prevention of Cardiovascular Diseases) Institute of Cardiovascular Research, Southwest Medical University, 646000 Luzhou, Sichuan, China; ^5^Nuclear Medicine and Molecular Imaging Key Laboratory of Sichuan Province, The Affiliated Hospital of Southwest Medical University, 646000 Luzhou, Sichuan, China

**Keywords:** PET/CT, abdominal aortic aneurysm, molecular mechanism, molecular imaging, review

## Abstract

Abdominal aortic aneurysm (AAA) is the most common and critical aortic disease. Bleeding is the most serious complication from a ruptured AAA, which often results in death. Therefore, early diagnosis and treatment are the only effective means to reduce AAA associated mortality. Positron emission tomography/computed tomography (PET/CT) combines functional and anatomical imaging. The expanded application of PET/CT in the medical field could have benefits for the diagnosis and treatment of patients with AAA. This review explores the efficiency of PET/CT in the diagnosis of AAA based on our understanding of the underlying molecular mechanisms of AAA development.

## 1. Introduction

Abdominal aortic aneurysm (AAA) is defined as a localized or extensive dilation of the abdominal aorta. Specifically, an increase in diameter of more than 50% is considered an AAA. The occurrence of AAA is related to multiple factors, such as age, gender, genetics, inflammation, and arteriosclerosis [1]. At present, apart from surgery, there is no particularly effective method for treating AAA [2]. Therefore, early detection is necessary to prevent the occurrence of ruptured abdominal aortic aneurysm (RAAA). Positron emission tomography/computed tomography (PET/CT) is a full-body imaging technique that, can quickly generate both functional and anatomical images. Therefore, PET/CT can be used to obtain a comprehensive and accurate diagnosis, and the advent of this imaging technology has been beneficial for diagnostic medicine [3, 4]. PET/CT is widely applied in the diagnosis and treatment of various diseases in clinical practice [5, 6]. PET/CT has been shown to have unique value for diagnosing vascular diseases as well [7]. This review summarizes the literature on the pathogenesis of AAA and the application of PET/CT in the diagnosis and treatment of AAA.

## 2. AAA

### 2.1 Background of AAA

Although some countries or regions have reported a decrease in the incidence of AAA over the past few decades, the specific reasons are unclear [8]. However, the risk of AAA should not be underestimated, and it is particularly important to screen specific populations that have been reported to be at higher risk for AAA (including seniors, males, long-term smokers, and those with a family history of AAA) [9, 10]. Once AAA is diagnosed, a reasonable management and treatment plan is required. Based on current guidelines, AAAs with diameters greater than 5.5 cm (5.0 cm for women) usually require surgical treatment, and those with diameters less than 4.0 cm can be monitored for changes in aneurysm size through follow-up examinations [11]. However, whether or not to surgically treat aneurysms between 4.0 cm–5.5 cm remains controversial [12]. Therefore, effective monitoring of AAA is extremely important for these patients.

### 2.2 Biology and Pathogenesis

AAA is a complex and multifactorial disease with genetic and environmental risks. Multiple studies have confirmed that the pathogenesis of AAA is mainly related to the infiltration of inflammatory cells, degradation of extracellular matrix (ECM), biological changes of vascular smooth muscle cells (VSMCs) and angiogenesis [13, 14]. Inflammatory factors can promote the development of AAA through the innate immune system and immunoglobulin mediated release. Chromosomal genetic changes may also show the same result, for example, the absence of Alpha1-antitrypsin will increase the level of plasma inflammatory molecules involved in AAA lesions. The phenotypic differences determined by genetics are 70–80%, and common environmental influences account for 20–30% (such as infection, smoking, or occupational exposure). The promoter of SMCs in AAAs is partially hypomethylate, leading to reduced vascular structural stability and increased inflammation, promoting the AAA phenotype [15, 16, 17]. In addition to the above biological changes, there is evidence that increased mechanical pressure on the longitudinal wall caused by aortic segmental sclerosis can also promote aneurysm growth [18].

**Fig. 1.**
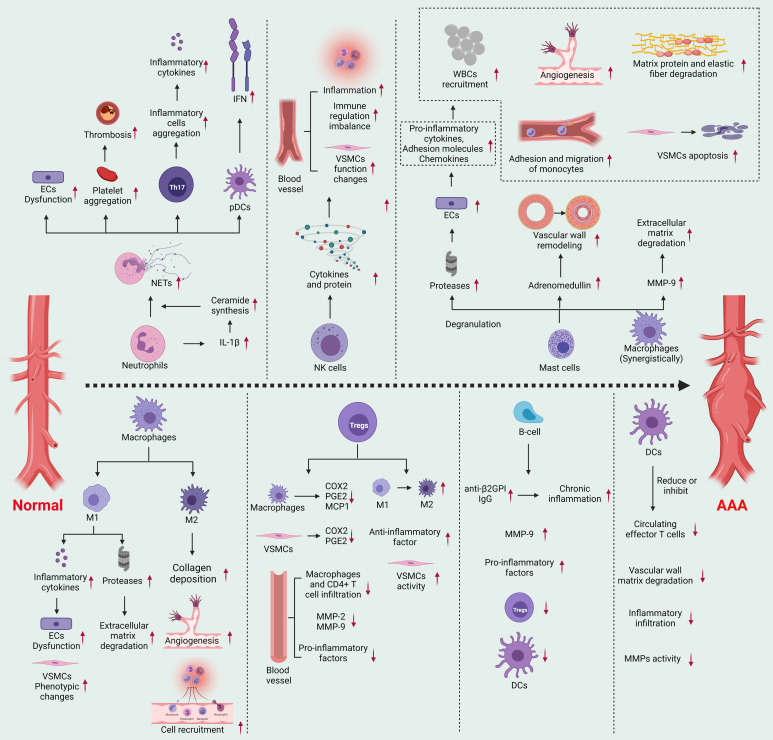
**Molecular mechanism of AAA caused by inflammatory immune cells**. Created with BioRender.com.

Although the molecular mechanism of AAA pathogenesis is not fully understood. Inflammation is still considered to be a central factor in the development of AAA. Under the stimulation of pathological factors, the expression of various inflammatory cells in the aortic wall will increase. Inflammatory cells can secrete a large number of proteases, which in turn degrade the ECM of the middle membrane and destroy the defense of the inner and outer membranes. The disruption of the vessel wall structure induces the entry of multiple mediators (such as neutrophils, cytokines, proteases and reactive oxygen species) into the vessel wall, creating an inflammatory microenvironment. They interact to form an inflammatory microenvironment, which in turn participates in the occurrence and development of AAA [19, 20, 21]. Human AAA biopsy reports suggest that AAA may be a T-cell specific antigen-mediated immune disease, which further supports the hypothesis that AAA-associated inflammation is a response [13]. Therefore, inflammation and immune cells play an important role in the formation and development of AAA (Fig. 1).

#### 2.2.1 Macrophages

Macrophages are key components of inflammatory processes and are mainly divided into two subtypes (M1 and M2) [22]. Studies have shown that M1-type macrophages can release various inflammatory cytokines, such as tumor necrosis factor-α (TNF-α), interleukin (IL)-6, IL-12, IL-1β, IL-18, chemokine (CC motif) ligand 2, monoxide nitrogen (NO), interferon (IFN), and reactive oxygen species (ROS) [23, 24]. These inflammatory factors aggravate the local inflammatory response in the aortic wall, as well as promoting endothelial cells (ECs) dysfunction and VSMC phenotypic differentiation, both of which underlie the formation and development of AAA [25, 26]. Activated M1 macrophages also secrete a variety of proteases, such as matrix metalloproteinase (MMP) and cathepsin, which are involved in ECM degradation in the vascular wall, which promotes remodeling of the abdominal aortic wall [27, 28, 29]. In contrast, M2 macrophages can prevent the progressive dilation and wall remodeling of AAA by reducing inflammation and promoting tissue repair [22, 30, 31]. M2 macrophages can also cooperate with mast cells and natural killer (NK) cells to promote angiogenesis, cell recruitment and collagen deposition [32]. An imbalance in the M1/M2 ratio can thus impact the formation and progression of AAA [33]. As such, targeting activation of M2 macrophages may be beneficial to reduce chronic inflammation associated with AAA formation.

#### 2.2.2 Neutrophils

Neutrophils are one of the most important effector cells in the immune response. Their primary functions include phagocytosis, degranulation, and formation of neutrophils extracellular traps (NETs) [34]. NETs involve a complex reticular fiber structure composed of chromatin, DNA, histones and various enzymes (including elastase, catheter protease and myeloperoxidase) [35]. The protease in the NETs can cause EC dysfunction and direct damage to the aortic wall [36, 37]. NETs may also promote thrombosis by enhancing platelet aggregation [38, 39]. In addition, NETs can induce T-helper (Th) 17 cell differentiation and recruit more inflammatory cells to the developing AAA via increasing IL-6 and pre-IL-1β transcription in macrophages [35]. In the elastase-induced AAA model, neutrophil deficiency has been shown to slow down the expansion of AAA in an MMP-independent manner, but the specific mechanism remains unclear [40]. Yan *et al*. [41] showed that activation of neutrophil proteinase promoted the release of NETs, which in turn activated plasmacytoid dendritic cells (pDCs) and promoted the production of type I IFN, ultimately leading to AAA dilation in the elastinase-induced model. Upon stimulation, IL-1β produced by neutrophils can promote the synthesis of ceramide, which in turn increases the formation of NETs, leading to AAA progression. Inhibiting NETs formation can thus potentially also mitigate AAA formation [42].

#### 2.2.3 Lymphocytes

As an important cellular component of the immune response, lymphocytes have immune recognition function. Studies have shown that lymphocytes can directly affect the formation and progression of AAA by releasing cytokines, proteases, and other related factors [21, 43, 44]. Total lymphocyte defects can weaken angiotensin II (Ang Ⅱ)-induced atherosclerosis, but have not been shown to affect AAA formation and dilation [45].

2.2.3.1 Regulatory T Cells (Tregs)

Extensive clinical and basic studies have shown that Tregs play a protective role in the formation of AAA by regulating endogenous immune responses [46, 47, 48]. *In vitro* studies have shown that Tregs can reduce the expression of cyclooxygenase 2 and prostaglandin E2 in macrophages and VSMCs, increase VSMC activity and induce macrophage differentiation from M1 to M2, thus reducing the occurrence of AAA in Ang Ⅱ-induced models [49, 50]. CD4+ T cells are the major inflammatory infiltrating cells in human AAA tissues [43, 51]. Extensive research has confirmed that Tregs can reduce infiltration of macrophages and CD4+ T cells into the vascular wall, as well as reduce the expression of pro-inflammatory cytokines, enhance the production of anti-inflammatory factors, reduce the secretion of monocyte chemotactic protein-1 (MCP-1), and reduce the expression and activity of MMP-2 and MMP-9, thereby inhibiting AAA formation in response to Ang Ⅱ stimulation [52, 53]. However, selective depletion of Tregs can cause an inflammatory cell imbalance, which in turn can increase the susceptibility of aneurysms in some animal models [54].

2.2.3.2 NK and NK T Cells

Both NK and NK T cells are important immune cells that are involved in the regulation of cardiovascular diseases [55]. Numerous clinical studies have confirmed that NK and NK T cells are widely present in human atherosclerotic AAA tissues [56, 57] and they can induce inflammation, immune cell imbalance, and functional changes of VSMCs by promoting the production of various cytokines and proteins, thereby contributing to AAA formation [58, 59, 60, 61]. α-Galactosylceramide (α-Galcer) is a very effective NK T cell agonist, which can be effectively combined with cluster of differentiation 1d (CD1d) [62]. Studies have shown that α-Galcer can promote a higher incidence of AAA in an Ang II-induced model, and pathological examination confirmed that inflammatory cell infiltration and pathological changes were significantly increased in this model [63]. The underlying mechanism may involve activation of NK T cells and increased expression of matrix degrading enzymes in VSMCs and macrophages [64]. However, Saito *et al*. [65] reported contrasting findings. Their study showed that α-Galcer can induce M2 polarization by activating NK T cells, weakening vascular endothelial-mediated AAA formation in an ob/ob mouse model. These contradictory results may be due to differences in animal models. Therefore, more in-depth studies are needed to better understand the role of NK and NK T cells in AAA formation.

2.2.3.3 B Lymphocytes

B lymphocytes are derived from hematopoietic stem cells in the bone marrow. Studies have shown that overactivated B cells may increase the secretion of antibodies, such as anti-β2GPI IgG, which can induce chronic inflammation and promote AAA formation [66]. In animal models of AAA, B cell defects have been shown to inhibit AAA formation and progression, which may be related to a decrease in spleen tyrosine kinase (Syk) activation and MMP-9 expression [67], or due to an increase in plasma cell like dendritic cells (DCs), resulting in an increase in Tregs and a decrease pro-inflammatory factors [68]. However, as the dominant B cell subtype in animal models of AAA, B2 cells can promote an increase in Tregs through, thus inhibiting AAA formation [69].

#### 2.2.4 Mast Cells

Mast cells are a kind of pro-inflammatory cell that is widely distributed around in vessels. Mast cells are involved in immune cell regulation, cell homeostasis, and cytokine secretion [70, 71]. Mast cells can release a variety of proteases (such as chymase and tryptase) through degranulation, thereby inducing expression of various pro-inflammatory cytokines (IL-6 and IFN-γ) [72] and adhesion molecules and chemokines (CCR2) [73] in ECs. This results recruitment of white blood cells, adhesion and migration of monocytes, VSMC apoptosis, ECM degradation, and angiogenesis [74, 75]. Mast cells can also cause vascular wall remodeling by releasing adrenomedullin [76], and by synergistically increasing in the activity of MMP-9 produced by monocytes and macrophages [77], which in turn contributes to AAA pathology. While inhibition of mast cell protease secretion has shown to be effective in preventing and treating AAA [78], some studies have reported that inhibiting mast cells are not protective against AAA development [79, 80]. Therefore, further studies are needed to confirm whether targeting mast cells is beneficial for treating AAA.

#### 2.2.5 DCs

DCs are phagocytotic and antigen presenting cells that can influence AAA pathology [81, 82]. Studies have shown that blocking DCs can inhibit the occurrence of AAA by reducing circulating effector T cells and inhibiting ECM degradation in the vascular wall [83, 84]. Kajimoto *et al*. [85] confirmed that atorvastatin can inhibit DCs, thereby reducing inflammatory cell infiltration and MMP activation in the vascular wall, thus inhibiting AAA occurrence and expansion.

#### 2.2.6 Matrix Metalloproteinases

MMPs are a class of proteolytic enzymes with similar structures that require metal ions as cofactors. Under the stimulation of pathological factors, ECs, neutrophils, macrophages and SMCs in the vascular wall can produce various types of MMPs, thus degrading the extracellular matrix [86]. In addition, genetic factors can also affect the expression of MMPs, thus increasing the risk of AAA [87]. Plenty of existing literature reports show that the MMPs mainly affect the occurrence and development of AAA, including MMP-1, -2, -3, -9, -12 and -13 [88]. Abnormal activation and expression of MMPs can not only affect the formation and progression of AAA, but also be used to evaluate the risk of AAA rupture [89, 90] and predict the occurrence of endoleaks after endovascular aortic repair (EVAR) [91]. At the same time, MMPs may also affect AAA by regulating angiogenesis and the phenotypic and functional changes of SMCs [92].

In summary, many inflammatory cells and factors contribute to AAA formation and development (Table 1, Ref. [35, 36, 37, 38, 39, 40, 41, 42, 43, 44, 47, 49, 50, 54, 56, 57, 67, 68, 70, 71, 77, 78, 79, 80, 87, 88, 89, 93, 94, 95, 96, 97, 98, 99, 100, 101, 102, 103]).

**Table 1.** **Inflammatory immune cells and AAA**.

Cell	Mechanism	Effect on AAA	References
Macrophages			
M1	① Release various inflammatory cytokines, promote the dysfunction of ECs and the phenotypic changes of VSMCs.	Positive	[35, 36, 37, 38, 39, 40, 41]
	② Secrete a variety of proteases.		[42, 43, 44]
M2	Cooperate with mast cells and NK cells to promote angiogenesis, cell recruitment and collagen deposition.	Negative	[47]
Neutrophils	① Promote thrombosis by enhancing platelet aggregation.	Positive	[49, 54]
	② Induce Th17 cell differentiation and recruit more inflammatory cells by increasing IL-6 and pre-IL-1β transcription in macrophages.		[50]
	③ Activate pDCs, thereby promoting the production of IFN.		[56]
	④ The production of IL-1β can promote the synthesis of ceramide, which in turn leads to an increase in the formation of NETs.		[57]
Lymphocytes			
Tregs	① Reduce the expression of COX2 and PGE2 in macrophages and VSMCs, increase the activity of VSMCs, and induce the transformation of macrophages from M1 type to M2 type.	Negative	[67, 68]
	② Reduce the infiltration of vascular wall macrophages and CD4+ T cells, reduce the expression of pro-inflammatory cytokines, enhance the production of anti-inflammatory factors, reduce the secretion of macrophages MCP-1, and reduce the expression and activity of MMP-2 and MMP-9.		[70, 71]
NK and NK T cells	Promote the production of various cytokines and protein expression, and induce inflammation, imbalance of immune regulation and functional changes of VSMCs.	Positive	[77, 78, 79, 80]
B lymphocytes	① Overactivated B cells may increase the secretion of pathological antibodies(anti-β 2GPI IgG), which induce chronic inflammation.	Positive	[87]
	② B cell defects may inhibit the activation of Syk and reduce the expression of MMP-9; it may also increase the expression of DCs, which in turn leads to an increase in Tregs and a decrease in the expression of pro-inflammatory genes.		[88, 89]
Mast cells	① Through the release of proteases, the expression of a variety of pro-inflammatory cytokines, adhesion molecules and chemokines can be induced, which can promote leukocyte recruitment, monocyte adhesion and migration, VSMCs apoptosis, matrix degradation and angiogenesis.	Positive	[93, 94, 95, 96, 97, 98]
	② Release adrenomedullin, thereby synergistically promoting the increase of MMP-9 activity produced by monocytes and macrophages, causing vascular wall remodeling.		[99, 100]
DCs	After inhibiting or depleting DCs, it can inhibit the inflammatory infiltration of the blood vessel wall, reduce the expression of circulating effector T cells, and reduce the activity of MMP.	Positive	[101, 102, 103]

AAA, abdominal aortic aneurysm; ECs, endothelial cells; VSMCs, vascular smooth muscle cells; pDCs, plasmacytoid dendritic cells; DCs, dendritic cells; IFN, interferon; NETs, neutrophils extracellular traps; Tregs, regulatory T cells; MCP-1, monocyte chemotactic protein 1; NK, natural killer cell; COX2, cyclooxygenase 2; PGE2, prostaglandin E2; MMP, matrix metalloproteinase.

## 3. Molecular Imaging Targets

AAA formation and progression are the result of the interaction of various cytokines and cells [13]. The distribution and dose-effect relationship of different cytokines, combined with PET/CT imaging, can be used to understand the biological changes of AAA [104, 105]. The PET imaging agents commonly used in clinical practice include 18F-Fluorodeoxyglucose (18F-FDG) and Sodium Fluoride (18F-NaF) [93]. Based on the inflammatory pathological basis of AAA, 18F-FDG has been widely used to assess the degree of inflammation in the aneurysm [94]. However, 18F-NaF can image the deposition of molecular calcium during the formation of calcified plaque in arteries [95]. As summarized above, inflammation is critical in AAA occurrence and development. Extensive research has reported that multiple tracers (such as 64Cu-DOTA-ECL1I, 18F-FMCH, 68GA-DOTATATE, 11C-PK11195, GE180, and cFLFLF) can be specifically combined with corresponding targets to evaluate the degree of inflammatory cell infiltration, thus providing objective imaging reference indicators in the prognosis of AAA [96, 97, 98]. Angiogenesis is also an important pathological marker in AAA progression. Previous studies have shown that CD105 and integrin can be found in new blood vessels where αvβ3 is highly expressed, while 64Cu-NOTA-TRC105-Fab, 18F-FPPRGD2, 18F-fluidide, and 68Ga-RGD can specifically bind to their corresponding ligands, indirectly reflecting the degree of angiogenesis [99, 100, 106, 107]. 18F-3’-deoxy-3’-fluoro-L-thymidine (18F-FLT) can also be used to mark cell proliferation in AAA expansion [108]. Although a variety of tracers have been used in AAA prognosis in clinical and animal models (Table 2, Ref. [109, 110, 111, 112, 113, 114, 115, 116, 117, 118]), there is still a lack of highly specific and sensitive tracers to diagnose and evaluate AAA progression.

**Table 2.** **Tracking characteristics of different tracers in arteriosclerosis and AAA**.

Tracers	Molecular imaging targets	Diagnostic value or significance	Disease	References
18F-FDG	GLUT	Assess inflammation	AAA	[109]
18F-NaF	Microcalcification	Detection of microcalcifications in blood vessel walls	AAA	[110]
64Cu-DOTA-ECL1I	CCR2	Assess inflammation	AAA, ASO	[111]
18F-FMCH	Choline receptor	Assess inflammation	ASO	[112]
68GA-DOTATATE	SSTRs	Assess inflammation	ASO	[112]
11C-PK11195	TSPO	Assess inflammation	ASO	[112]
GE180	TSPO	Assess inflammation	ASO	[112]
cFLFLF	FPR1	Assess inflammation	ASO	[113]
64Cu-NOTA-TRC105-Fab	CD105	Understand angiogenesis	AAA	[114]
18F-FPPRGD2	αvβ3	Assess inflammation and understand angiogenesis	AAA	[115]
18F-Fluidide	αvβ3	Understand angiogenesis	AAA	[116]
68Ga-RGD	αvβ3	Understand angiogenesis	AAA	[117]
18F-FLT	TK-1	Understand cell proliferation	AAA	[118]

ASO, atherosclerosis; AAA, abdominal aortic aneurysm; 18F-FDG, 18F-Fluorodeoxyglucose; 18F-NaF, 18F-Sodium Fluoride; 18F-FMCH, 18F-Fluoro-Methyl Choline; cFLFLF, Cinnamoyl-F-(D) L-F-(D) L-F-K; GLUT, glucose Transporters; CCR2, chemokine receptor 2; SSTRs, somatostatin receptors; TSPO, translocator protein; FPR1, formyl peptide receptor 1; MMPs, matrix metalloproteinases; TK-1, thymidine kinase-1.

Abnormal activation and overexpression of MMPs may lead to ECM remodeling. Molecular imaging can track MMP expression and activity and thus be used an index of disease progression [119, 120]. Widely used MMP inhibitors (MMPIs) such as TPPTS, ^111^In-DTPA-RP782, ^123^I-HO-CGS 27023A [101], 18F-BR-351 [102], 18F-BR420 [103, 121], ^111^In-RP782 11a, ^99^mTc-RP805 11b [120], and other tracers can specifically bind to MMPs. Some tracers can even identify special subtypes or activated forms of MMPs, improving the disease diagnoses. With advancements in molecular imaging technology and targeted therapy, some MMPIs have been developed as new therapeutic drugs for clinical application [122]. Studies have shown that chloramphenicol can specifically bind to MMP-2. Based on these characteristics, labeling chloramphenicol with 18F-fluoropropionyl-chlorotoxin (18F-FP-chlorotoxin) has good diagnostic value for glioma [123]. 68Ga-DOTA-TCTP-1 also has good visibility for MMPs in inflammatory atherosclerotic lesions [124]. In conclusion, numerous MMP-based tracers have been applied in the diagnosis and treatment of tumors, arteriosclerosis and other diseases (Table 3, Ref. [125, 126, 127, 128, 129, 130, 131]). However, there have been few studies on the application of these tracers in AAA.

**Table 3.** **Application of MMP-related tracers**.

Tracers	Biological behaviors	Disease	Model	References
TPPTS	MMP activity	MI, ASO, Aneurysm	Mouse	[126]
^111^In-DTPA-RP782		ASO		
^123^I-HO-CGS 27023A		ASO		
18F-BR-351	MMP activity	Stroke, Colorectal cancer	Mouse	[127, 129]
F-BR420	MMP activity	ICD, Colorectal cancer	Mouse	[128, 129]
^111^In-RP782 11a	MMP activity	ASO	Mouse	[125]
^99^mTc-RP805 11b				
18F-FP-chlorotoxin	MMP-2 activity	Glioma	Mouse	[130]
68Ga-DOTA-TCTP-1	MMP activity	ASO	Mouse	[131]

TPPTS, 99mTc-Hydrazinonicotinyl-Tyr3-octreotide; 18F-FP-chlorotoxin, 18F-fluoropropionyl-chlorotoxin; MMPs, matrix metalloproteinases; MI, myocardial infarction; ASO, atherosclerosis; ICD, irritant contact dermatitis.

## 4. Animal Studies of AAA

Various animal models have been established to investigate the pathogenesis of AAA [132, 133]. Emerging molecular imaging tools, including ultrasound (US), magnetic resonance imaging (MRI), and PET have been widely used to research the molecular mechanism in experimental AAA animal models [134]. Based on the understanding that macrophages disrupt ECM stability in the arterial wall, Nahrendorf *et al*. [135] used nanoparticle labeled 18F to quantify macrophage accumulation in a mouse model of AAA. After applying a fluoride-labeled tracer, they used PET/CT imaging to evaluate cell proliferation, vascular inflammation and angiogenesis [100, 108].

αvβ3 is a transmembrane heterodimer integrin that connects the ECM to the cytoskeleton. Its natural ligands contain an arginine-glycine aspartic acid (RGD) sequence that binds high affinity [136, 137]. Kitagawa *et al*. [100] used 18F-labeled RGD and PET to study AAA in animal models and found changes in the degree of inflammation and angiogenesis. In addition to 18F, 68Ga labeled RGD derivatives have been studied of tumor angiogenesis [107]. The αVβ3 selective tracer 18F-fluciclatide has also been used to study angiogenesis in the wall of AAA [106].

## 5. Human and Patient Work

### 5.1 Assessing Aneurysmal Inflammation

The demand for imaging technology has advanced to include anatomical or structural imaging. For clinicians, the ability to determine biological changes in cells through functional imaging of molecules will further their understanding of the etiology and pathogenesis of disease [138, 139]. PET/CT can reveal metabolic activity by tracking the uptake of 18F-FDG in all cells and tissues that metabolize glucose. Although it is conventional knowledge that aneurysms area pathological manifestation of atherosclerosis, in-depth study of the underlying molecular mechanism of the aneurysm pathology could identify unique degenerative changes in the aortic vessel wall [140, 141]. Many existing studies have confirmed that pathological changes in molecular mechanisms that occur during AAA formation can be tracked with contrast agents and functional imaging, allowing one to predict disease development and future clinical events [19, 94, 96, 99, 100, 135].

Based on the pathological basis of AAA and the theoretical basis of functional imaging of PET/CT, the application of PET/CT in AAA detection and prognosis is increasing. Maximum FDG uptake is significantly related to the pathological characteristics and clinical symptoms of the aortic wall, including the degree of inflammatory cell infiltration, increased MMP expression, and plaque instability. Therefore, FDG-PET/CT imaging may improve risk prediction of AAA rupture [109]. McBride *et al*. [110] performed PET/CT and T2-weighted MRI on 15 asymptomatic AAAs before and 24 hours after ultrasmall superparamagnetic iron oxide (USPIO) administration and identified FDG-PET/CT and USPIO-MRI uptake of AAA-related vascular inflammation. Although there is little correlation between the two, the uptake of cell glycogen and distribution of phagocytic activity increased with significant differences in the lesion area. The analysis showed that 18F-FDG-mediated uptake by glucose transporters (GLUTs) in the inflammatory cells in the AAA wall, indicative of increased metabolic activity. However, there was no significant difference in FDG uptake in areas of severe calcification [105]. In contrast, PET/CT examination of the AAA wall of asymptomatic chronic inflammation with different tracers showed no increase of metabolic activity [111, 112].

Infected AAAs are often the result of bacterial or monilial infection of the abdominal aorta. Compared with atherosclerotic AAAs, they tend to increase sharply and rupture easily, and are not often diagnosed early. Clinical diagnosis and treatment of infected AAAs require bacterial blood cultures and clinical evidence of inflammation and morphological findings in a CT [113, 114]. However, reports have shown that PET/CT has significant value in the diagnosis of infected AAAs [115, 116]. Studies have shown a significant increase in the uptake of 18-FDG PET/CT in infected AAAs compared with non-infected AAAs. 18-FDG PET/CT can detect changes in AAAs and surrounding structures and provide reliable support for monitoring the AAA following treatment [117, 118].

Macrophages tracers have been developed and used to detect and monitor cardiovascular diseases. Studies have shown that macrophage activation can lead to increased expression of translocator protein [142], somatostatin receptor [125], and other proteins, as well as increase choline uptake [126]. Although selective tracers for these proteins have been used in studies of atherosclerotic diseases, their application in the diagnosis of AAA has not been studied [97]. Therefore, relevant tracers should be explored and applied in AAA.

### 5.2 Predicts AAA Growth and Clinical Outcomes

The use of 18F-NaF uptake for the evaluation of active vascular calcification in high-risk atherosclerotic plaques has shown initial success. Studies have shown that 18-NaF uptake was significantly increased in the aneurysm wall compared with non-aneurysm areas, and this increase was limited to areas with aneurysm disease and active calcification. The higher the 18F-NaF uptake, the faster the aneurysm can expand, indicative of a greater the possibility of aneurysm rupture and surgical repair. These results confirmed that 18-NaF PET/CT may be an objective indicator of AAA disease, aneurysm growth, and clinical events [127]. Nchimi *et al*. [128] used PET/CT to study the relationship between biomechanical characteristics and biological activity of AAA. The results showed that increased uptake of 18F-FDG PET in aneurysms was closely related to aneurysm wall stress, and risk factors, such as acquired and genetic sensitivity. For small AAAs, studies have shown that 18F-FDG uptake was low, likely due to a reduction in the number of cells capable of taking up 18F-FDG. However, the global level of 18F-FDG uptake is low, when the diameter exceeds the maximum AAA diameter [129, 143]. The specific cause for this inverse correlation is unclear. It is speculated, however, that when the diameter of the aneurysm is small, chronic inflammation is too low to detection an increase in glucose metabolism by the PET camera; and when the diameter of the aneurysm is increased, the thrombus metabolic activity in the AAA is enhanced. This can be accompanied by the production of various cytokines and proteases in the aneurysm wall, which may affect the metabolism and structure of the arterial wall, leading to an increase in glucose metabolism [111, 130].

Kotze and colleagues [131] consecutively recruited 34 patients with AAA for routine ultrasound examination and 18F-FDG PET/CT monitoring. During the follow-up period, nine patients were excluded from the study because they did not complete the 12-month follow-up. Preliminary results from a longitudinal observational study of 25 patients showed that patients with lower uptake of 18F-FDG may be more likely to develop AAA expansion in the future. However, in another well-controlled large cohort, there was no difference in average 18F-FDG tracer uptake between infra-renal AAA and normal aorta using SUV or TBR, and there no difference in visual intake scores. These finding demonstrated that metabolic activity varies widely and is independent of aortic diameter [144].

### 5.3 Prediction of Aneurysm Rupture Risk

AAAs are almost asymptomatic until they rupture. However, ruptured AAAs can cause catastrophic consequences for patients. Although CT can clearly diagnose the size of an AAA, aneurysm diameter alone cannot reliably identify high-risk AAAs; thus, better risk stratification is required [145]. Many studies have shown that AAA is a disease related to inflammatory cell infiltration, matrix protein degradation, and VSMC proliferation and apoptosis [59, 146, 147]. These pathological and molecular changes affect the AAA wall structure and induce expansion and rupture of the AAA [89, 148]. PET/CT can assess the extent of inflammatory cell infiltration through functional imaging, thereby predicting the risk of aneurysm expansion and rupture [149, 150]. Sakalihasan *et al*. [149] further demonstrated a possible association between increased uptake of 18-FDG in the aneurysm level and distention and rupture of AAAs using PET imaging of 10 patients. Thus PET/CT and 18-FDG are useful tools for assessing the risk of AAA rupture.

Due to constant changes in hemodynamics, the AAA wall can show uneven expansion, increasing the likelihood of AAA rupture [150, 151, 152]. Extensive research has confirmed that when the AAA wall is under high mechanical stress, especially when there is significant intramural thrombosis, metabolism accelerates and 18F-FDG uptake increases. Therefore, the combination of PET imaging and wall stress analysis can more determine the relationship between biomechanical changes due to hemodynamics, remodeling of the lumen, and inflammation, which may provide a more reliable prediction for the risk of aneurysm rupture [128, 149, 153]. 64Cu-DoTA-ECL1I was used to track the expression of CCR2 in the aneurysm wall of AAA in an elastase-induced AAA rat model. The results showed that the tracer uptake in the ruptured AAA was significantly higher than in the non-ruptured AAA [96]. Therefore, the CCR2 tracer 64Cu-DoTA-ECL1I has clinical value for predicting the AAA rupture risk.

However, Marini *et al*. [154], proposed that AAAs are the result of a multi-factor processes characterized by the gradual loss of cell populations associated with irreversible remodeling of the aortic connective tissue, ultimately leading to aneurysm rupture. When the lumen diameter is relatively large, the cell density in the vessel wall decreases to a very low level, and the positive index of a PET/CT scan is relatively low. In fact, with an increase in AAA diameter, there is a significant loss of cell and tissue structure within the diseased wall that increased the risk of rupture caused by mechanical stressors.

The surgical indications of AAA are mainly based on color Doppler ultrasound or CT to assess the aneurysm diameter. However, AAA growth is non-linear, and AAAs of any diameter are at risk of rupture. Recent research claims that relying only on the diameter of the AAA to determine surgical treatment is not accurate. For clinicians, understanding the various risk factors other than AAA size alone is important for early and appropriate intervention for aneurysm repair. Further research on the stratification factors for predicting AAA rupture is needed to provide theoretical support for treatment [97, 155].

### 5.4 Post Procedural Complications and Their Evaluation

At present, the main treatment for AAAs is EVAR, but various complications can occur during or after surgery. The most common complication after EVAR is endoleak, which is mainly caused by the relationship between the graft itself and the anatomy of the aneurysm. The structural and morphological changes of the graft and the infection of the graft can also lead to postoperative complications. Systemic complications mainly include end-organ ischemia, cardiovascular and cerebrovascular events, and post-implantation syndrome [156]. However, endoleak is a key factor affecting long-term outcomes. The persistence of large endoleaks indicates EVAR failure. Therefore, timely detection and treatment of endoleak are particularly important [157]. PET/CT can predict the occurrence of endoleaks after EVAR, providing a reliable basis for the early detection and diagnosis of postoperative endoleaks [158, 159, 160]. Graft infection is the most serious complication after covered stent repair of AAA. PET/CT has been used to determine postoperative graft infection. However, the surface of synthetic graft materials may cause chronic inflammatory response after being implanted in the body, and uptake may be increased after PET/CT with 18F-FDG [161, 162]. Due to the risk of false positives, the use of PET/CT in the diagnosis of graft infection needs to be carefully evaluated in combination with relevant laboratory and imaging examinations [163]. Marie *et al*. [164], analyzed FDG uptake with performing PET/CT scans after EVAR. The results showed that PET/CT had guiding value for understanding the changes of aneurysm body. At present, there are few reports on the evaluation of PET/CT after EVAR, some of which include case reports. Therefore, more prospective studies are needed in this field.

**Fig. 2.**
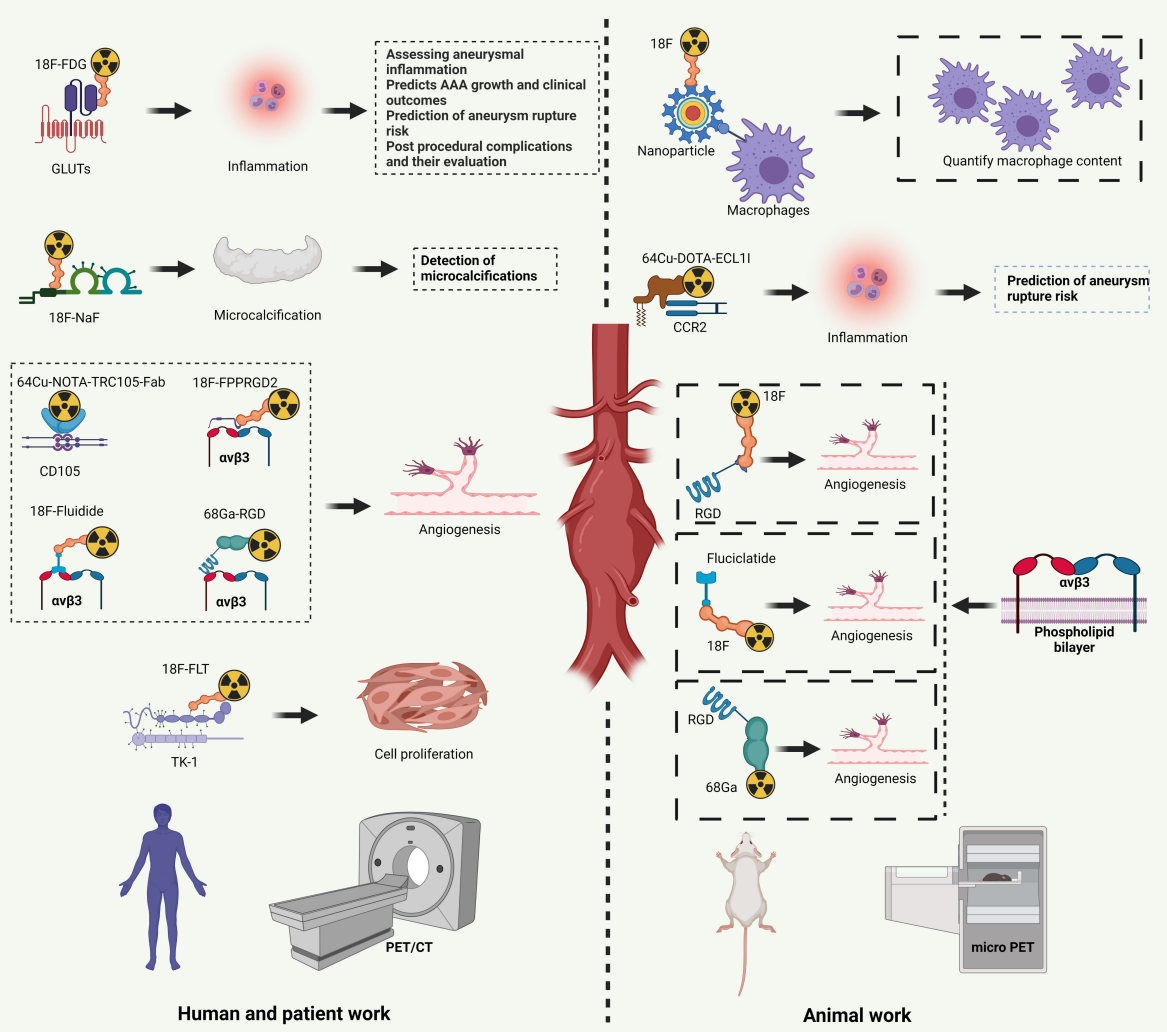
**Application value and significance of different tracers in human AAA**. Created with BioRender.com.

In conclusion, although tracers targeting different targets have shown unique advantages in evaluating AAA occurrence and development in human and animal studies, they each have certain limitations (Fig. 2). Therefore, it is necessary to search for specific markers for AAA and develop more reliable tracers.

## 6. Summary and Prospective

AAA is one of the most common vascular diseases. It is a major burden on global health care and poses a huge challenge to global public health. Early prevention, diagnosis and management of AAAs are particularly important. PET/CT has been shown to be of great significance in clinical diagnosis of diseases, including cardiovascular disease. PET/CT can be used to localize and quantify metabolic activity of inflammatory cells in an aneurysm. 18-FDG combined with PET/CT is a complementary imaging method that can be used in the diagnosis and follow-up of aortic pathologies associated with inflammatory aneurysm and aortic infection, including mycotic AAAs, infected prostheses, and stent grafts. Therefore, multi-center, large-sample, high-quality prospective studies are needed to realize the transformation of PET/CT with tracers from preclinical research to clinical research, thereby expanding the ability to diagnose and treat AAA.
